# Gray matter structural alterations in first-episode drug-naïve adolescents with major depressive disorder: a comprehensive morphological analysis study

**DOI:** 10.1017/S0033291725000790

**Published:** 2025-04-11

**Authors:** Baoshuai Zhang, Baolin Wu, Xun Zhang, Hongsheng Xie, Yanxin Ling, Ziru Zhao, Ruoqiu Gan, Lihua Qiu, Andrea Mechelli, Zhiyun Jia, Qiyong Gong

**Affiliations:** 1 Department of Radiology, Huaxi MR Research Center (HMRRC), Institute of Radiology and Medical Imaging, Functional and Molecular Imaging Key Laboratory of Sichuan Province, West China Hospital of Sichuan University, Chengdu, China; 2 Research Unit of Psychoradiology, Chinese Academy of Medical Sciences, Chengdu, China; 3 Department of Nuclear Medicine, West China Hospital of Sichuan University, Chengdu, China; 4 Medical Imaging Center, The Second People’s Hospital of Yibin, Yibin, China; 5 Department of Psychosis Studies, Institute of Psychiatry, Psychology & Neuroscience, King’s College London, London, UK; 6 Xiamen Key Laboratory of Psychoradiology and Neuromodulation, Department of Radiology, West China Xiamen Hospital of Sichuan University, Xiamen, China

**Keywords:** adolescent major depressive disorder, deformation-based morphometry, psychoradiology, surface-based morphometry, voxel-based morphometry

## Abstract

**Background:**

Major depressive disorder (MDD) tends to emerge during adolescence; however, neurobiological research in adolescents has lagged behind that in adults. This study aimed to characterize gray matter (GM) structural alterations in adolescents with MDD using comprehensive morphological analyses.

**Methods:**

This study included 93 adolescent MDD patients and 77 healthy controls. Voxel-based morphometry (VBM), deformation-based morphometry (DBM), and surface-based morphometry (SBM) methods were used to analyze GM morphological alterations in adolescent MDD patients. Sex-by-group and age-by-group interactions, as well as the relationships between altered GM structure and clinical characteristics were also analyzed.

**Results:**

Whole-brain VBM and DBM analyses revealed GM atrophy in the left thalamus and bilateral midbrain in adolescent MDD patients. Whole-brain SBM analysis revealed that adolescent MDD patients, relative to controls, showed decreased thickness in the left postcentral gyrus and left precentral gyrus; increased thickness in the bilateral superior temporal gyrus, left parahippocampal gyrus and right lateral orbitofrontal gyrus; and decreased fractal dimension in the right lateral occipital gyrus. A significant sex-by-group interaction effect was found in the fractal dimension of the left lateral occipital gyrus. The volume of the left thalamus and the thickness of the left superior temporal gyrus were correlated with the duration of disease in adolescent MDD patients.

**Conclusions:**

This study suggested that adolescent MDD had GM morphological abnormalities in the frontal-limbic, subcortical, perceptual network and midbrain regions, with some morphological abnormalities associated with disease duration and sex differences. These findings provide new insight into the neuroanatomical substrates underlying adolescent MDD.

## Introduction

Major depressive disorder (MDD) is a common mental disorder characterized by core symptoms of persistent depressed mood and loss of pleasure or interest in daily activities. Adolescence (ages 15–19) represents a critical risk factor for MDD, with a prevalence rate of ~2.14% (Marx et al., 2023). This developmental stage is marked by significant structural and functional changes in the brain, which are essential for the maturation of cognitive and emotional processes (Chu et al., 2018). However, this developmental window also increased the vulnerability to psychiatric disorders, especially during the COVID-19 pandemic (Ambrosetti et al., 2021). The ongoing processes of synaptic pruning and myelination during adolescence may result in distinct structural vulnerabilities in the developing brain (Gogtay & Thompson, 2010). Compared with adult MDD patients, adolescent patients often exhibit poorer responses to pharmacological therapies (Marx et al., 2023). Moreover, adolescent-onset MDD can have profound and lasting effects on individuals’ psychological health and social functioning (Blank et al., 2022). Additionally, the precise treatment of adolescent MDD also faces challenges, primarily due to nonadherence among patients, with the severity of depression being one of the main predictors of nonadherence (Pompili et al., 2013). Overall, these considerations underscore the importance of the need to better understand the neurobiology of adolescent MDD.

Structural magnetic resonance imaging (MRI) is a powerful neuroimaging tool that detects gray matter (GM) anatomical changes associated with psychiatric disorders. Based on high-resolution brain anatomical images, voxel-based morphometry (VBM), deformation-based morphometry (DBM), and surface-based morphometry (SBM) are commonly used to quantitatively analyze brain morphological abnormalities in patients. VBM and DBM can be used to assess GM volume changes at the voxel level, while DBM is more sensitive to the atrophy of subcortical areas and anatomical information in the deformation field (Borghammer et al., 2010). Unlike VBM and DBM, which assess the GM volume information, SBM is widely used to examine cortical characteristics such as cortical thickness, sulcal depth, surface area, and gyrification. The combination of these morphological analyses enables quantitative assessment of brain volume, shape, and surface features, providing critical insights into the structural changes that occur during adolescent brain development, which is characterized by significant neurodevelopmental processes such as synaptic pruning and myelination. Structural MRI studies have consistently reported that adult MDD patients exhibit GM structural abnormalities in the frontal, subcortical, and limbic regions (Zhang, Peng, Sweeney, Jia, & Gong, 2018). However, neuroimaging research in adolescents has lagged behind that in adults. Although some studies have attempted to reveal GM structural changes in adolescents with MDD (Qiu et al., 2023; Hagan et al., 2015; Mo et al., 2023; Pannekoek et al., 2014; Redlich et al., 2018; Shad, Muddasani, & Rao, 2012; Straub et al., 2019; Wehry et al., 2015), the results are inconsistent. A possible explanation is that some of these studies involved participants taking different types and doses of antidepressant medication, which is known to affect brain structure (Serafini, 2012). More importantly, previous studies have predominantly used VBM analysis to identify changes in GM volume in adolescent MDD patients. However, VBM may not be able to fully detect GM structural abnormalities associated with adolescent MDD. Considering that the VBM, DBM, and SBM analyses reflect different morphological characteristics of the GM, the combination of the three methods may provide a more comprehensive understanding of changes in brain structure in adolescents with MDD.

Thus, the main purpose of the present study was to comprehensively characterize the patterns of change in GM structure in adolescent MDD patients using combined VBM, DBM, and SBM analysis. To rule out the effects of psychotropic medication, only first-episode drug-naive adolescent MDD patients were included in our study. We hypothesized that similar to adult MDD patients, adolescent MDD patients should exhibit abnormal GM volume or cortical shape in brain regions involved in emotion processing and cognitive functions.

## Methods

### Participants

This study was approved by the Research Ethics Committee of West China Hospital, Sichuan University. Written informed consent was obtained from all participants and their parents or legal guardians. Adolescent MDD patients and healthy adolescents aged 12–18 years were recruited. The diagnosis of depression was determined by two experienced clinical psychiatrists according to the criteria of the DSM-V. The severity of depression was rated using the 17-item Hamilton Rating Scale for Depression (HAMD-17). The HAMD-17 is a widely used tool for measuring the severity of depressive symptoms. It consists of 17 items that evaluate various aspects of MDD, with higher total scores indicating greater severity of MDD. Adolescents with MDD were included if they met a diagnosis of first-episode MDD and were drug-naive, with a total HAMD score ≥18. Healthy adolescents were included if they and their first-degree relatives had no current or past MDD diagnoses. The exclusion criteria for both groups were as follows: (1) had a history of neurological diseases or severe head injury; (2) had a history of chronic medical conditions; (3) had a history of other psychiatric disorders or brain developmental disorders; and (4) had contraindications for MRI scans. Finally, 93 adolescent MDD patients (45 males and 48 females; mean age 15.96 ± 1.65 years) and 77 healthy adolescents (37 males and 40 females; mean age 16.11 ± 1.60 years) were included.

### MRI data acquisition

All participants underwent high-resolution T1-weighted anatomical MR imaging on a 3.0-T Discovery MR750 scanner (GE Healthcare, Milwaukee, WI) with the following parameters: repetition time, 8.2 ms; echo time, 3.2 ms; inversion time, 450 ms; field of view, 256 × 256 mm^2^; matrix size, 256 × 256; voxel size, 1.0 × 1.0 × 1.0 mm^3^; flip angle, 12°; and 188 sagittal slices covering the whole brain, with a thickness of 1.0 mm without a gap.

### Data preprocessing and analysis

The structural MRI data were preprocessed and analyzed using the Computational Anatomy Toolbox version 12 (CAT12, https://neuro-jena.github.io/cat/index.html) (Gaser et al., 2023), an extension toolkit of the Statistical Parametric Mapping version 12 (SPM12, https://www.fil.ion.ucl.ac.uk/spm/software/spm12). Data preprocessing and all analyses were performed using the default parameters described in the CAT12 manual.

#### VBM analysis

First, for better registration, we manually reoriented the high-resolution T1-weighted anatomical images to the anterior commissure in SPM12. Second, the default ICBM tissue probability map (TPM) in SPM12 was used to segment the high-resolution structural images into GM, white matter (WM), cerebrospinal fluid, bone, nonbrain soft tissue, and background. Third, diffeomorphic anatomical registration through exponentiated lie algebra (DARTEL) (Ashburner, 2007) in SPM12 was used for morphological and anatomical registration, normalization, and modulation. The GM images were aligned and resampled to 1.5 × 1.5 × 1.5 mm^3^ and then normalized to the Montreal Neurological Institute (MNI152) space. The inverse Jacobian matrix of local transformation was used to modulate the segmented GM to retain the volume measurement. Finally, the normalized and modulated GM images were smoothed using an 8-mm full-width at half-maximum (FWHM) Gaussian kernel. Additionally, the total intracranial volume (TIV) was calculated for each participant.

#### DBM analysis

In DBM analysis, the segmented GM images were nonlinearly registered to the MNI template. Then, DBM (i.e. the determinant of the Jacobian transformation matrix) maps were calculated to estimate the local volume in each voxel (DBM values). Positive values indicate increased DBM indices (i.e. tissue enlargement) and negative values indicate decreased DBM indices (i.e. tissue atrophy). Finally, the Jacobian determinants were resampled to 1.5 × 1.5 × 1.5 mm^3^ and smoothed using a 6-mm FWHM Gaussian kernel.

#### SBM analysis

Briefly, high-resolution T1-weighted MR images underwent tissue segmentation to estimate the WM distance. Local maxima were then projected to other GM voxels by using a neighbor relationship described by the WM distance. These values are equal to the cortical thickness. This projection-based method also includes partial volume correction, sulcal blurring, and sulcal asymmetries without sulcus reconstruction. A topological correction was performed using a spherical harmonics-based approach. An adapted volume-based DARTEL algorithm was applied to the surface for spherical registration. For group comparisons, the cortical thickness maps of the left and right hemispheres were reparameterized into a common coordinate system and smoothed with a 12-mm FWHM Gaussian kernel. Additionally, the gyrification, sulcus depth and cortical complexity (i.e. fractal dimension) were also calculated based on the central surfaces, and then smoothed using a 25-mm FWHM Gaussian kernel as recommended by CAT12.

### Statistical analysis

The differences in the clinical and demographic data between the two groups were analyzed using SPSS v26.0 software (IBM Corp, Armonk, NY). Two-sample *t* tests (two-tailed) were used to detect differences in age, education, body mass index (BMI) and HAMD score between the two groups, and chi-squared tests were used to detect gender ratio differences between the two groups.

The between-group differences in GM morphological parameters were assessed with two-sample *t* tests. The thresholds were set as voxel-wise *p* < 0.001 and cluster-wise *p* < 0.05 (family-wise error [FWE] corrected) for VBM and DBM analyses. For SBM analysis, the threshold was set as vertex-wise *p* < 0.001 and cluster-wise *p* < 0.05 (FWE corrected). A general linear model was used to investigate potential age-by-group and sex-by-group interaction effects on GM morphology, using the same statistical thresholds as above. For regions that showed significant between-group differences, partial correlation analysis was used to examine the correlation between their mean values and clinical characteristics, including age of onset, illness duration and HAMD score. Age and gender were set as covariates in all analyses, and TIV was also used as a covariate in the VBM and DBM analyses.

## Results

### Demographic and clinical characteristics

There were no significant differences in gender (*p* = 0.965), age (*p* = 0.551), education level (*p* = 0.631) or BMI (*p* = 0.935) between adolescent MDD patients and HCs. For adolescent MDD patients, the mean illness course was 10.13 ± 4.98 months, the mean age of onset was 15.28 ± 1.76 years, and the mean HAMD-17 score was 22.74 ± 6.06. The detailed demographic and clinical characteristics of the two groups are shown in Table 1.

**Table 1.** Demographic and clinical characteristics of the participants

	MDD patients (*n* = 93)	Healthy controls (*n* = 77)	*p*-Value
Gender (male/female)	45/48	37/40	0.965
Age (years)	15.96 ± 1.65	16.11 ± 1.60	0.551
Education (years)	8.95 ± 1.64	9.07 ± 1.57	0.631
BMI (kg/m^2^)	21.44 ± 2.45	21.41 ± 1.79	0.935
Disease duration (months)	10.13 ± 4.98	–	–
Age of onset (years)	15.28 ± 1.76	–	–
HAMD score	22.74 ± 6.06	2.58 ± 1.17	<0.001

*Note*: All quantitative data are expressed as mean ± standard deviation; numbers for gender data. MDD, major depressive disorder; BMI, body mass index; HAMD, Hamilton Depression Rating Scale.

### GM morphological alterations in adolescent MDD patients

**Figure 1.**
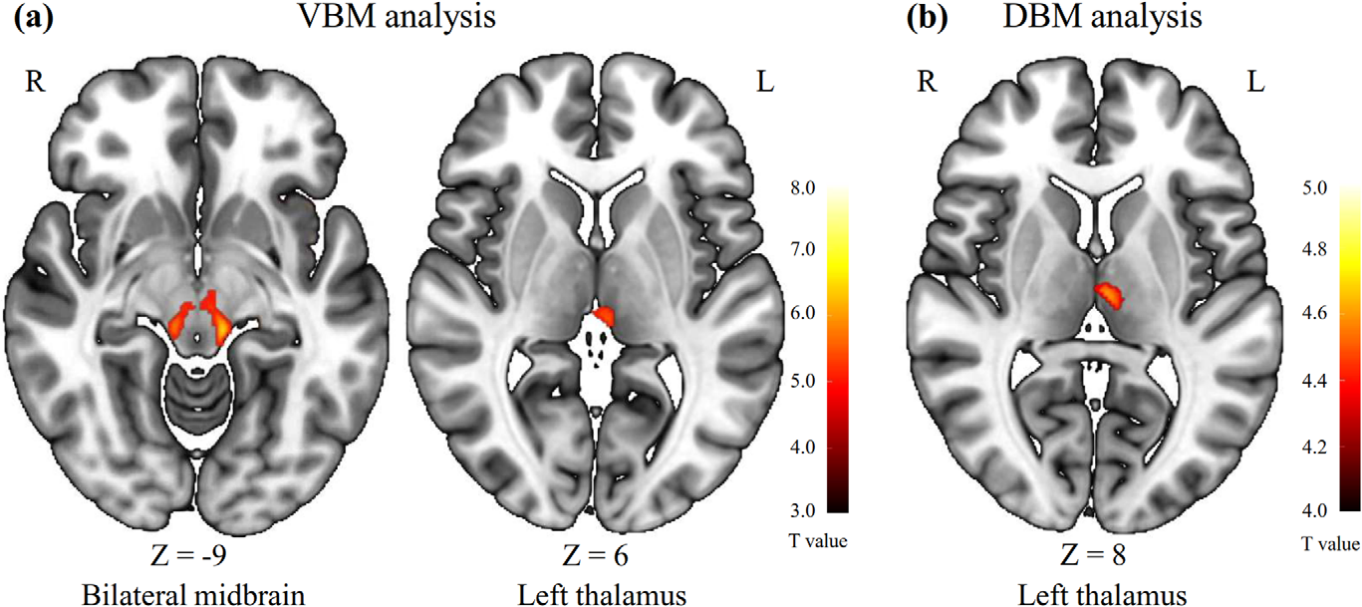
Brain regions showing significantly decreased gray matter volume in adolescent major depressive disorder patients compared with healthy controls according to the VBM (a) and DBM (b) analyses. The color bar represents the T statistics. VBM, ‘voxel-based morphometry’; DBM, ‘deformation-based morphometry’; R, ‘right’; L, ‘left’.

According to the whole-brain VBM analysis, compared with HCs, adolescent MDD patients showed significant GM atrophy in the bilateral midbrain and left thalamus (Table 2 and Figure 1a). According to the whole-brain DBM analysis, compared with HCs, adolescent MDD patients had significant less GM volume in the left thalamus (Table 2 and Figure 1b).

**Table 2.** Brain regions showing abnormal gray matter volume in MDD patients compared with HCs in the whole-brain VBM and DBM analysis

	Brain region	Side	Peak MNI coordinates			Cluster size (voxels)	Peak intensity	*p*-Value
			*x*	*y*	*z*			
VBM analysis								
* HC > MDD*	Midbrain	L	−8	−35	−3	609	6.70	<0.001
	Midbrain	R	8	35	−3	288	6.22	<0.001
	Thalamus	L	3	−3	11	170	5.44	0.003
* HC < MDD*	None							
DBM analysis								
*HC > MDD*	Thalamus	L	−5	−17	6	101	4.63	0.019
* HC < MDD*	None							

*Note*: Statistical analyses of VBM and DBM were performed based on a whole-brain level (voxel-wise uncorrected *p* < 0.001 and cluster-wise *p* < 0.05, family-wise error corrected). MDD, major depressive disorder; HC, healthy control; MNI, Montreal Neurological Institute; VBM, voxel-based morphometry; DBM, deformation-based morphometry; R, right; L, left.

**Figure 2.**
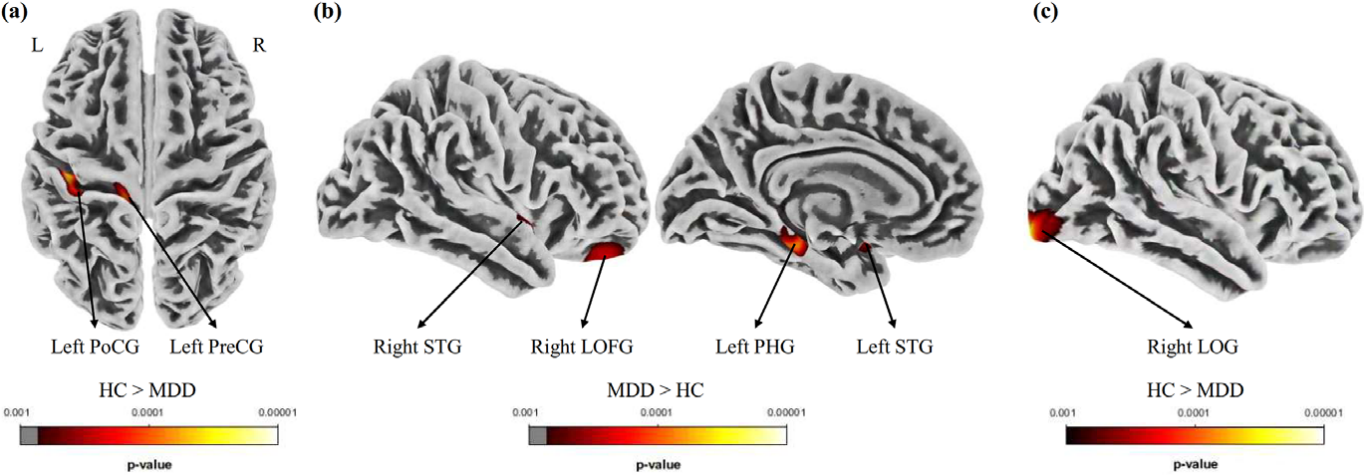
Brain regions showing significantly altered cortical thickness (a, b) and fractal dimension (c) in adolescent major depressive disorder (MDD) patients compared with healthy controls (HC) according to the surface-based morphometry analysis. The color bar represents *p* statistics. PoCG, ‘postcentral gyrus’; PreCG, ‘precentral gyrus’; STG, ‘superior temporal gyrus’; LOFG, ‘lateral orbitofrontal gyrus’; PHG, ‘parahippocampal gyrus’; LOG, ‘lateral occipital gyrus’; R, ‘right’; L, ‘left’.

According to the whole-brain SBM analysis, compared with HCs, adolescent MDD patients exhibited significantly decreased thickness in the left postcentral gyrus and left precentral gyrus; significantly increased thickness in the bilateral superior temporal gyrus, left parahippocampal gyrus and right lateral orbitofrontal gyrus; and significantly lower fractal dimension in the right lateral occipital gyrus. There were no significant differences in sulcus depth or gyrification between the two groups. The detailed results of the SBM analysis are shown in Table 3 and Figure 2.

**Table 3.** Brain regions showing significantly altered cortical morphology in adolescent MDD patients compared with HCs in the whole-brain SBM analysis

	Brain region	Side	Peak MNI coordinates			Cluster size (vertices)	Peak intensity	*p*-Value
			*x*	*y*	*z*			
Thickness								
* HC > MDD*	Postcentral gyrus	L	–43	–22	52	912	4.45	<0.001
	Precentral gyrus	L	−12	−33	61	465	4.19	0.031
* HC < MDD*	Superior temporal gyrus	L	−51	−1	−8	732	4.62	0.002
	Parahippocampal gyrus	L	−20	−30	−19	478	4.18	0.027
	Superior temporal gyrus	R	−48	−9	−10	504	3.82	0.021
	Lateral orbitofrontal gyrus	R	15	46	−24	520	3.67	0.018
Fractal dimension								
* HC > MDD*	Lateral occipital gyrus	R	19	−101	−12	189	4.25	0.039
* HC < MDD*	None							

*Note*: Statistical analyses of cortical thickness and fractal dimension were performed based on a whole-brain level (vertex-wise *p* < 0.001 and cluster-wise *p* < 0.05, family-wise error corrected). MDD, major depressive disorder; HC, healthy control; MNI, Montreal Neurological Institute; SBM, surface-based morphometry; R, right; L, left.

### Interaction analysis results

**Figure 3.**
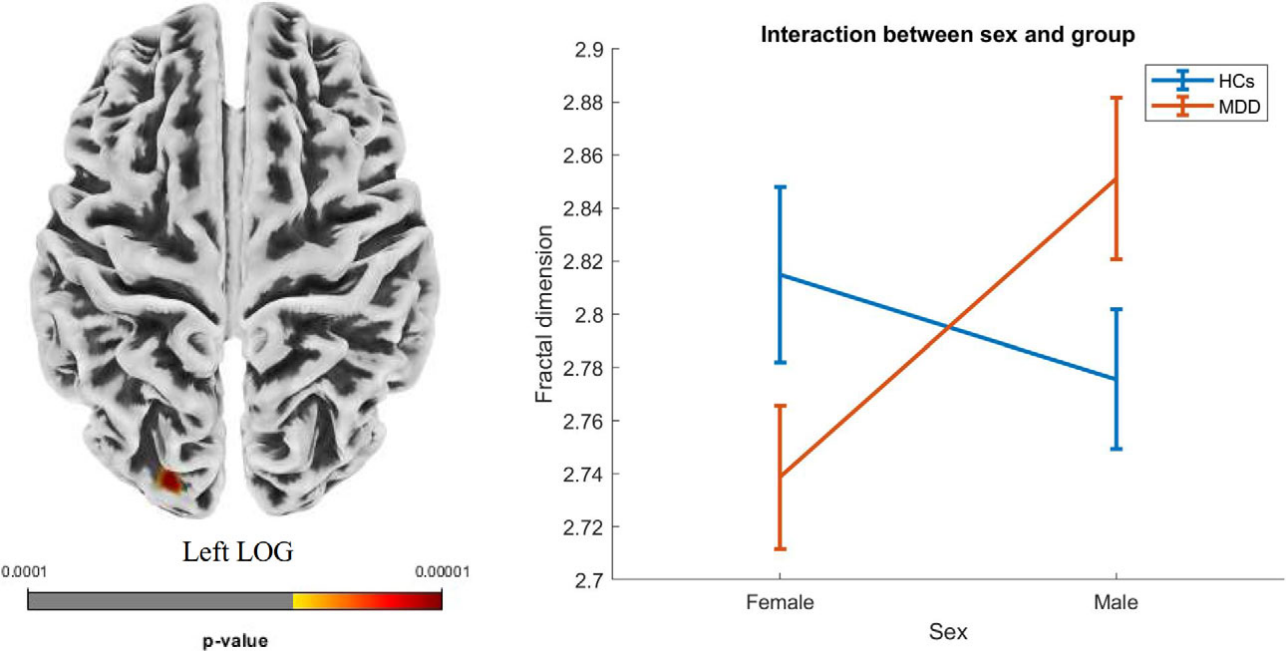
Brain regions showing significant sex-by-group interaction effect. Female adolescent major depressive disorder (MDD) patients showed lower fractal dimension in the left lateral occipital gyrus (LOG) compared with female healthy controls (HCs), while male adolescent MDD patients showed higher fractal dimension in the same region compared with male HCs.

A significant sex-by-group interaction effect was found in the fractal dimension of the left lateral occipital gyrus (peak MNI coordinates: *x* = −21, *y* = −92, *z* = 15; cluster size = 31; peak intensity = 4.28; *p* = 0.016). Specifically, female adolescent MDD patients showed a significantly lower fractal dimension in the left lateral occipital gyrus compared with female HCs, whereas male adolescent MDD patients exhibited a significantly higher fractal dimension in the same region compared with male HCs (Figure 3). No significant age-by-group interaction effects were found.

### Correlation analysis results

**Figure 4.**
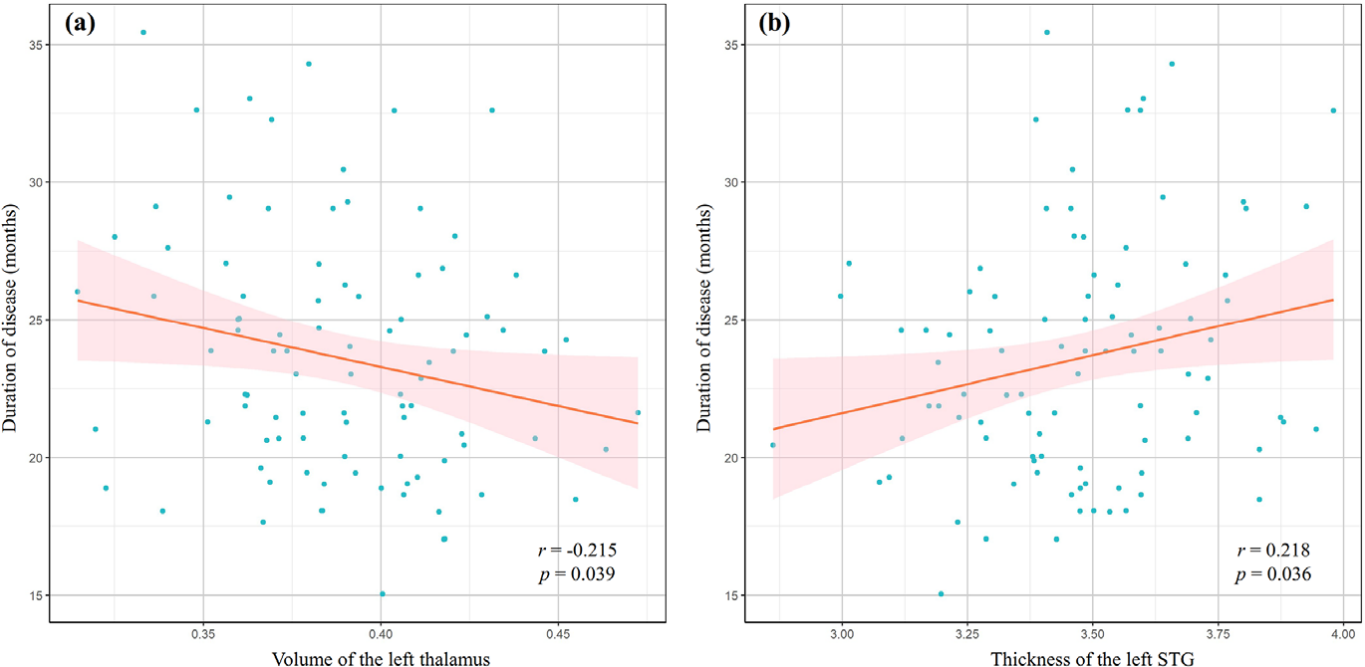
Partial correlation analyses show that the duration of disease is negatively correlated with the volume of the left thalamus (a) and positively correlated with the thickness of the left superior temporal gyrus (STG) (b) in adolescents with major depressive disorder.

Figure 4 shows the correlations between altered GM morphological parameters and clinical characteristics in adolescents with MDD. Specifically, the GM volume of the left thalamus was negatively correlated with duration of disease (*r* = −0.215, *p* = 0.039, uncorrected) (Figure 4a). There was a significant positive correlation between the thickness of the left superior temporal gyrus and duration of disease (*r* = 0.218, *p* = 0.036, uncorrected) (Figure 4b).

## Discussion

The main findings of our study were as follows: (1) the VBM and DBM analyses revealed GM volume abnormalities in the left thalamus and bilateral midbrain in adolescent MDD patients; (2) the SBM analysis suggested that adolescent MDD patients had abnormal cortical morphology in the frontal-limbic, visual, sensorimotor and auditory network regions; and (3) morphological alterations of the left thalamus and left superior temporal gyrus correlated with duration of disease in adolescent MDD patients. Furthermore, a significant sex-by-group interaction effect was identified in the fractal dimension of the left lateral occipital gyrus.

To our knowledge, this study is the first to characterize GM structural alterations in a first-episode drug-naive sample of adolescent MDD patients by using multiple morphological analyses. By including only first-episode drug-naive patients, we gain valuable insights into the neuroanatomical changes associated with the initial onset of adolescent MDD, effectively minimizing the confounding effects of pharmacotherapy that can obscure these changes in individuals who have undergone treatment. Unlike previous studies that focused on a single morphological analysis (Schmaal et al., 2017), the integrated use of three analytical strategies in our study allowed us to better characterize the GM structural abnormalities associated with adolescent MDD by assess multiple morphological characteristics. The VBM, DBM and SBM analyses suggest that GM morphological abnormalities in adolescent MDD patients are expressed at multiple levels, including volumetric deficits in subcortical structures crucial for emotional processing and cortical abnormalities in regions associated with higher cognitive functions and emotional regulation. Thus, the combination of VBM, DBM, and SBM analyses might provide distinct yet complementary insights into the neuroanatomical substrates underlying adolescent MDD.

Adolescent MDD-related GM volume deficits were found in the left thalamus and bilateral midbrain. A previous meta-analysis of VBM studies also confirmed decreased thalamic volume in adults with MDD (Du et al., 2012). Abnormalities in cortical shape, WM microstructure, nodal centralities and metabolism have also been observed in the left thalamus among individuals with MDD (de Diego-Adeliño et al., 2014; Ende, Demirakca, & Tost, 2006; Lu et al., 2016; Wu et al., 2023). The thalamus is an important subcortical structure and is considered a complicated sensory information node that controls emotion, memory, and arousal (Wolff, Alcaraz, Marchand, & Coutureau, 2015). Moreover, a prior study revealed that deep brain stimulation of the thalamus could achieve antidepressant effects (Drobisz & Damborská, 2019), suggesting that the thalamus may act as an optimal target for treating depression. Our recent diffusion tensor imaging study identified the left thalamus as a hub node in brain structural networks (Wu et al., 2024), underscoring its crucial role in facilitating interactions within the brain networks. Therefore, regional GM volume reduction in the left thalamus may impact its functional communication with other brain regions. The midbrain is associated with emotional responses and cognitive control, and dopamine neurotransmitters within the midbrain are closely related to the addictiveness of individuals to nonsuicidal self-injury behavior (Mayer, Kahl, Uzuneser, & Fendt, 2018). Similarly, a prior VBM study revealed decreased GM volume in the midbrain in late-onset MDD patients (Hwang et al., 2010). Decreased GM volume in the midbrain of adolescent MDD patients may be closely related to disturbances in the reward process. It is worth noting that GM atrophy in the left thalamus and bilateral midbrain has been rarely reported in previous whole-brain VBM studies, potentially due to factors such as medication usage. By ruling out the effects of psychotropic medication, our study highlights the important role of the left thalamus and bilateral midbrain in the neurobiology of adolescent MDD.

The SBM analysis revealed that adolescent MDD-related cortical morphological abnormalities were observed in the frontal and limbic regions, including the right lateral orbitofrontal cortex and left parahippocampal gyrus. Similarly, previous studies have demonstrated structural and functional abnormalities in the orbitofrontal cortex and parahippocampal gyrus in adolescents with MDD (Cheng et al., 2024; Schmaal et al., 2017; Villa et al., 2020; Wu, Li, Zhou, Zhang, & Long, 2020; Zhang et al., 2023; Zhou et al., 2022). Furthermore, cortical abnormalities in these brain regions may also lead to dysfunction of the frontal-limbic neural circuit, which is essential for regulating emotions and mood and is often disrupted in adults with MDD (F. F. Zhang et al., 2018). Impaired structural and functional connectivity in frontal-limbic neural circuits was also observed in adolescent MDD patients (Geng et al., 2016). Thus, our findings further support the notion that frontal-limbic regions play critical roles in the neurobiology of adolescent MDD.

In addition to the frontal-limbic regions, abnormal cortical morphology was also found in the visual (right lateral occipital gyrus), sensorimotor (left postcentral and left precentral gyrus) and auditory (bilateral superior temporal gyrus) network regions, which are involved in emotional processing and low-level cognitive functions. While this is not a common finding in the existing literature, some of the prior studies have reported abnormal GM volume, cortical morphology, and resting-state neural activity in the perceptual network regions in adolescents with MDD (Liu et al., 2022; Schmaal et al., 2017; X. Zhang et al., 2023; Zhou et al., 2022). Taken together, our findings suggest that cortical structural alterations of the visual, sensorimotor and auditory network regions may also play a role in the neurobiology of adolescent MDD and may explain impaired perceptual function.

In this study, we identified a significant sex-by-group interaction effect in the fractal dimension of the left lateral occipital gyrus. Previous study also revealed significant sex-by-group interaction effects on cortical morphometry in patients with MDD (Hu et al., 2022). Our results revealed intriguing differences between male and female adolescents with MDD in comparison to controls. As mentioned above, the lateral occipital gyrus is pivotal in visual perception and processing, indicating its potential role in how adolescents with MDD respond to visual stimuli and process emotional content. The decreased fractal dimension observed in female MDD patients may reflect structural or functional changes that impair visual processing or integration within emotional contexts. This reduction could represent a maladaptive neural mechanism associated with the higher rates of internalizing symptoms commonly observed in females with MDD. Conversely, the increased fractal dimension in male adolescents with MDD may suggest heightened neural complexity or enhanced processing capabilities in the left lateral occipital gyrus. This finding could indicate a compensatory response to emotional distress or a different pattern of visual processing that engenders resilience in certain contexts. Male adolescents with MDD may engage different cognitive strategies or neural pathways compared with their female counterparts, potentially resulting in divergent neurodevelopmental trajectories in response to depressive symptoms. These sex differences underscore the importance of considering biological and psychosocial factors when interpreting the neuroanatomy of MDD. The observed interaction effect implies that gender may play a critical role in determining the neurobiological underpinnings of MDD in adolescents. Therefore, it is essential for clinicians and researchers to recognize that treatment approaches may need to be tailored not only to the symptoms of MDD but also to the sex of the adolescent patient.

Moreover, we found that duration of disease was negatively correlated with the GM volume of the left thalamus. This finding suggests that as the duration of MDD increases, there may be a progressive loss of GM volume in the left thalamus. This decline could reflect underlying neurodegenerative processes or the result of prolonged psychological stress, which is known to adversely affect brain morphology. Such finding also highlights the importance of early intervention in adolescents with MDD, as timely treatment could potentially mitigate the detrimental effects on brain structure.

In addition, the present study also identified a positive association between the cortical thickness of the left superior temporal gyrus and duration of disease. This finding suggests that as the duration of MDD increases, there may be an associated increase in the structural thickness of this specific brain region. The observed increase in thickness might indicate neuroadaptive responses to the chronic stress associated with prolonged depressive episodes. It is possible that, in the face of ongoing psychological distress, individuals may develop compensatory neural mechanisms that enhance certain cognitive and emotional processing capabilities. This phenomenon, often referred to as neuroplasticity, suggests that the brain may respond dynamically to enduring challenges by reinforcing specific structural elements over time.

Several limitations should be acknowledged in our study. First, the current study utilized a cross-sectional design, which limits the ability to observe how GM structure may change over the course of adolescent MDD. Second, regarding the correlation analysis, we would like to clarify that the results did not survive after correction for multiple comparisons. This limitation underscores the need for cautious interpretation of the results and suggests that further research is warranted to confirm these findings in larger samples. Finally, while the study focuses on adolescents diagnosed with MDD, considerations should be given to how these findings might generalize to different populations, including younger children or adults with MDD, as similar structural patterns could potentially exist. Additional studies are needed to explore whether these GM abnormalities are present in other mood disorders or in varying severity levels of MDD across different age groups, which may help in understanding the broader applicability of the results.

## Conclusions

Using combined VBM, DBM and SBM analyses, the present study provided evidence that adolescent MDD patients exhibited significant GM morphological abnormalities across several key brain regions, specifically in the frontal-limbic, subcortical, perceptual network, and midbrain regions, which are critical for emotional processing and cognitive functions. Furthermore, some morphological abnormalities were associated with disease duration and sex differences. On the one hand, these findings may enhance our understanding of the neuroanatomical substrates underlying adolescent MDD, contributing to a more nuanced view of the disorder’s pathophysiology. On the other hand, the identified structural abnormalities may serve as potential biomarkers for early diagnosis and intervention strategies in adolescent MDD, aiding in the development of targeted treatments.
